# Predictors of Neurotoxicity in a Large Cohort of Italian Patients Undergoing Anti‐CD19 Chimeric Antigen Receptor (CAR) T‐Cell Therapy

**DOI:** 10.1002/brb3.70891

**Published:** 2025-09-23

**Authors:** Anna Modoni, Catello Vollono, Eugenio Galli, Luca Capriati, Federica Sorà, Stefan Hohaus, Serenella Servidei, Nicola Piccirillo, Paolo Calabresi, Simona Sica

**Affiliations:** ^1^ UOC Neurologia, Dipartimento di Neuroscienze, Organi di Senso e Torace Fondazione Policlinico Universitario Agostino Gemelli IRCCS Rome Italy; ^2^ Dipartimento Universitario di Neuroscienze Università Cattolica del Sacro Cuore Rome Italy; ^3^ UOC Neurofisiopatologia, Dipartimento di Neuroscienze, Organi di Senso e Torace Fondazione Policlinico Universitario Agostino Gemelli IRCCS Rome Italy; ^4^ Dipartimento Di Scienze di Laboratorio ed Ematologiche Fondazione Policlinico Universitario A. Gemelli IRCCS Rome Italy; ^5^ Sezione Di Ematologia, Dipartimento di Scienze Radiologiche Ed Ematologiche Università Cattolica del Sacro Cuore Rome Italy

**Keywords:** CAR‐T, EEG, GammaGT, ICANS, predictors of neurotoxicity

## Abstract

**Background:**

Anti‐CD19 chimeric antigen receptor (CAR) T‐cell therapy is an innovative and effective treatment for patients with B‐cell hematological malignancies. Despite its high efficacy, it has been associated with the development of acute toxicities that can be severe or even fatal. Indeed, cytokine release syndrome (CRS) and immune effector cell‐associated neurotoxicity syndrome (ICANS) can induce significant morbidity and require close monitoring. Identification of clinical and laboratory markers able to predict the occurrence of ICANS may allow prompt recognition and more effective management strategies.

**Methods:**

Here, we report a retrospective study on a cohort of 81 Italian adult patients treated in our hospital between September 2019 and April 2024. We reviewed all clinical, demographic, laboratory, and neurophysiological data in order to identify potential predictors.

**Results:**

The results of the multivariate analysis confirmed that ICANS typically occurred less frequently in younger patients, especially when treated with 41BB co‐stimulated CAR‐T. Baseline EEG abnormalities are confirmed to be a fundamental predictor of neurotoxicity. Interestingly, we identified GammaGT as a new, statistically significant marker of ICANS. This represents a novel finding, probably related to the important role of GammaGT also in neuroinflammation.

**Conclusions:**

Our results need to be confirmed in a larger cohort of patients in order to eventually be integrated into current clinical practice and management of patients undergoing CAR‐T.

## Graphical abstract

Immune effector cell–associated neurotoxicity syndrome (ICANS) is one of the most serious complications of CAR‐T cell therapy. Identifying biochemical and neurophysiological predictors of ICANS is crucial for developing effective prevention and management strategies. Multivariate analysis identified younger age as protective against ICANS (HR = 0.964, *p *= 0.047), while elevated gamma‐GT (HR = 1.026, *p *= 0.038) and baseline EEG slowing activity (HR = 9.335, *p *= 0.016) were significant predictors of ICANS development. These findings identified a subgroup of patients at particularly high risk, who may benefit from tailored clinical monitoring approaches.

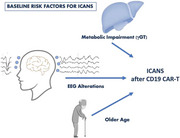

## 1 Background

Cancer immunotherapy has been a remarkable advancement in recent years, with Chimeric Antigen Receptor T‐cell (CAR‐T cell) therapy emerging as a revolutionary approach in the treatment of various hematological malignancies (Grant et al. 2022). This innovative approach involves genetically modifying a patient's T cells to express chimeric antigen receptors (CARs) that recognize specific antigens present on cancer cells. Upon infusion back into the patient, these engineered CAR‐T cells proliferate and mount a potent immune response against the targeted malignancy, leading to durable remissions and, in some cases, long‐lasting cures. (June et al. 2018) Notably, CAR‐T cell therapy has demonstrated remarkable efficacy in heavily pretreated hematological malignancies derived from B‐lymphocytes, such as acute B‐lymphoblastic leukemia (B‐ALL), diffuse large B‐cell lymphoma (DLBCL), follicular lymphoma (FL), mantle cell lymphoma (MCL), and more recently, multiple myeloma (MM). (Grant et al. 2022). Despite its therapeutic efficacy, CAR‐T cell immunotherapy has been extensively associated with the development of acute toxicities that can be severe or even fatal. CAR‐T cells can cause adverse events through two main mechanisms. The first mechanism is autoimmune toxicity, known as “on‐target, off‐tumor toxicity,” which occurs when CAR‐T lymphocytes recognize antigens expressed on healthy tissues. (Flugel et al. 2023) The second mechanism involves cytokine release‐related toxicity. This includes cytokine release syndrome (CRS), which in some cases can progress to macrophage activation syndrome, and probably neurological toxicity, known as immune effector cell‐associated neurotoxicity syndrome (ICANS) (Sterner and Sterner 2022). ICANS is a “frontal encephalopathy” usually emerging 1–3 weeks following CAR‐T cell infusion, though late onset occurs in approximately 10%–30% of cases (Neelapu et al. 2018), posing a significant clinical challenge that requires prompt recognition and management. This condition is the most feared complication of CAR‐T cell therapy, and it often accompanies and possibly correlates with CRS but can also manifest independently. (Gust et al. 2017) The pathophysiology of ICANS involves a complex interplay of immune dysregulation, cytokine storm, blood–brain barrier disruption, neuroinflammation, and neuronal dysfunction. (Siegler and Kenderian 2020) In more detail, the activation of endothelial cells, as evidenced by increased levels of Angiopoietin‐2 and Von Willebrand Factor, leads to the production of pro‐inflammatory factors such as VEGF and IL‐8. These contribute to further endothelial activation, resulting in increased vascular permeability and microthrombosis with disseminated intravascular coagulation (DIC), as well as the disruption of the blood–brain barrier (Wang et al. 2025). Interestingly, some bedside scores can be used to assess endothelial dysfunction, such as the modified EASIX. This score takes into account C‐reactive protein (CRP), LDH, and platelet (PLT) count, and has been reported to be useful in predicting ICANS occurrence (Galli et al. 2023). As a consequence of blood–brain barrier integrity disruption, CAR‐T cells, along with other immune cells and a large amount of pro‐inflammatory cytokines, migrate into the brain, causing neuronal dysfunction. Interestingly, CD19 antigen is also expressed by brain pericytes, mediating the so‐called “on‐target, off‐tumor toxicity” of CAR‐T cells (Flugel et al. 2023). Among the cytokines most involved in the pathogenesis of ICANS are not only IL‐1 and IL‐6, which also play a role in the development of CRS, but also IFN‐γ, IL‐10, and GM‐CSF, which contribute specifically to the development of ICANS (Wang et al. 2025). The incidence of ICANS varies widely, as it is related to the CAR‐T product, the dose of CAR‐T infused, the type of lymphodepleting chemotherapy, and disease characteristics. Risk factors for severe ICANS include CD28‐CAR‐T products, higher doses of CAR‐T, high disease burden, preexisting neurological conditions, thrombocytopenia, and early and/or severe CRS (Gust et al. 2017). Studies have reported ICANS rates ranging from 2% to 70% in patients receiving CAR‐T cells targeting either CD19 or BCMA antigens (Garcia Borrega et al. 2019), while a “class‐effect” neurotoxicity with an atypical Parkinsonism has been described only following CAR‐T cells targeting BCMA (Berdeja et al. 2021). Indeed, ICANS encompasses a spectrum of neurologic and psychiatric manifestations, ranging from mild cognitive impairment and confusion to severe encephalopathy, seizures, and cerebral edema. (Grant et al. 2022) Neuroimaging typically appears normal in cases with clinically confirmed ICANS. Nonetheless, MRI and CT scans can be useful for ruling out other conditions that present similar neurological symptoms. In recent years, several studies have identified electroencephalography (EEG)—a low‐cost, easily accessible bedside tool—as a sensitive predictor of neurotoxicity (Beuchat et al. 2022; Hernani et al. 2024; Pondrelli et al. 2025). In particular, qualitative EEG abnormalities observed prior to infusion have been proposed by some authors as potential risk factors for the development of ICANS (Hernani et al. 2024; Pondrelli et al. 2025). Conversely, post‐infusion EEG abnormalities have been reported to precede and predict the clinical onset of neurotoxicity, suggesting their potential role in early detection and monitoring (Hernani et al. 2024). Taken together, these data suggest that EEG assessment may not only have predictive value but also a potential role in guiding therapeutic decisions in patients with ICANS. Indeed, the management of ICANS encompasses a multidisciplinary approach, including pharmacologic interventions with steroids and IL‐1 inhibitor‐1. (Jain et al. 2023) The proper use of therapies is essential to balance the control of ICANS while preserving the antitumor efficacy of CAR‐T cells. The present study delves into the intricate realm of predictors of neurotoxicity in a large cohort of Italian patients undergoing CAR‐T cell immunotherapy. We retrospectively reviewed all clinical, demographic, laboratory, and neurophysiological data to identify potential predictors.

## 2 Methods

### 2.1 Cohort

Inclusion criteria: adult patients (aged 18 years and above) affected by B‐cell malignancies, specifically DLBCL, other large B‐cell lymphomas, and acute lymphoblastic leukemia (B‐ALL), who were eligible for CAR‐T treatment between September 2019 and April 2024, were consecutively included in this study. Exclusion criteria: patients affected by preexisting neurological conditions and/or other severe psychiatric or cardiovascular disorders, or any other illness that could affect data interpretation, were excluded. Written informed consent was obtained from each participant. The present study was performed in agreement with the Declaration of Helsinki and was approved by the local Ethics Committee (ID 4879 Prot 0020777/22 Amendment 09/2024).

### 2.2 CAR‐T Cell Procedures

A standard lymphodepleting chemotherapy regimen was administered in a hospital setting according to each commercial product's instructions, based on fludarabine/cyclophosphamide in all cases. CAR‐T products included Axicabtagene Ciloleucel (axi‐cel), Tisagenlecleucel (tisa‐cel), and Brexucabtagene Autoleucel (brexu‐cel), according to specific labels.

Bridging therapies included chemotherapy or radiotherapy or simple observation, according to the treating hematologist's decision. In all cases, patients did not receive intrathecal chemotherapy or brain irradiation as bridging therapy.

### 2.3 Biological Monitoring

Laboratory data at baseline and daily after CAR‐T infusion included complete blood count with differential, ferritin, alkaline phosphatase, liver function tests, renal function tests, lactate dehydrogenase (LDH), fibrinogen, CRP, and erythrocyte sedimentation rate (ESR). Furthermore, we included in the analysis inflammatory prognostic markers calculated on raw laboratory data, namely neutrophil to lymphocyte ratio (NLR), platelet to lymphocyte ratio (PLR), monocyte to lymphocyte ratio (MLR), neutrophil to monocyte ratio (NMR), systemic immune‐inflammation index (SII), and neutrophil to lymphocyte × platelet ratio (NLPR). Moreover, we included in the study the Endothelial Activation and Stress Index (EASIX), calculated with the following formula: (LDH [U/L] × creatinine [mg/dL]/PLTs [10^9^ cells/L]), and the more recent modified version of this score (m‐EASIX), which replaces creatinine with CRP (mg/dL) in the same formula. These markers were selected based on their relationship with endothelial damage as well as with outcomes in CAR‐T therapy‐related toxicities, according to clinical experience and available literature (Pennisi et al. 2021).

### 2.4 Neurophysiological Monitoring

Before CAR‐T infusion, a baseline standard laboratory 19‐lead EEG, including stimulation such as eyes open and closed as well as hyperventilation, was performed on Day 5 before CAR‐T infusion. This neurophysiological assessment was repeated upon clinical indication, when symptoms suggestive of ICANS occurred. EEG was performed bedside with a median latency of 2 h (range 1–3 h since development of first symptoms). In some cases, according to ICANS severity, EEG was systematically repeated daily in order to strictly monitor cerebral activity. All EEGs were evaluated by visual inspection without using automatic seizure detection software. Two neurologists with particular expertise in epileptology reviewed recordings daily. Discrepancies in interpretation were resolved through critical review and consensus. The background activity was assessed for predominant background EEG frequency, while EEG findings were categorized as follows: diffuse slow (theta and delta) activity, focal slow activity, focal to bilateral synchronous slow activity, focal epileptiform discharges, and diffuse epileptiform discharges.

### 2.5 Toxicity Assessments and Management

At the time of CAR‐T infusion, all patients started a prophylactic antiepileptic schedule with Levetiracetam 500 mg bid. After CAR‐T infusion, patients were closely monitored and evaluated for toxicity: CRS was scored based on the American Society for Transplantation and Cellular Therapy (ASTCT) criteria, assigning 0 to 5 points according to clinical relevance and the required pharmacological support (Lee et al. 2019). This procedure was carried out three times daily or whenever there was a change in the patient's status. Finally, we reported the timing of ICANS onset and the ICANS severity score using the ASTCT criteria, which includes the ICE scores, together with evaluations for awakeness, cognitive and superior functions, dysgraphia, or seizures (Lee et al. 2019). The management of patients developing CRS included fluidotherapy, oxygen administration, and IL‐6 inhibitors, according to the clinical assessment as reported above. ICANS management included high doses of dexamethasone infusion, according to ICE scores and according to current guidelines (Hayden et al. 2022).

### 2.6 Statistical Analyses

To evaluate the predictive factors of neurotoxicity, we categorized patients with ICANS scores ≥ 1 as ICANS+. Other patients were categorized as ICANS−. We analyzed and compared clinical and neurophysiological data between the ICANS+ and ICANS− groups.

Numeric variables (age, complete blood count with differential, ferritin, alkaline phosphatase, liver function tests, renal function tests, LDH, fibrinogen, CRP, ESR, EASIX, mEASIX, CRS score, CRS timing of symptom onset, and PDR) were compared using either one‐way analysis of variance or the Mann–Whitney *U*‐test, depending on data distribution. A multivariable analysis was conducted among variables associated with ICANS. All numeric variables were analyzed as continuous variables, and an ROC analysis was applied if any variable was found to be associated with ICANS in multivariate analysis in order to identify a cut‐off.

Nominal variables (sex, race, diagnosis, comorbidities, number and type of previous treatments, EEG abnormalities, and CRS‐ and ICANS‐related symptoms) were analyzed and compared between ICANS+ and ICANS− groups using 2 × 2 contingency tables, with either the chi‐square test or Fisher's exact test as appropriate. Univariate and multivariate logistic regression analyses were performed to identify risk factors for ICANS. The level of statistical significance was set at *p* < 0.05. ROC analysis was applied to identify a cut‐off for continuous variables. All statistical analyses were conducted using SPSS and NCSS software.

## 3 Results

### 3.1 Cohort Description

A total of 81 patients were enrolled (M/F: 46/35; mean age: 56.20 ± 13.03). Among them, six patients (7.4%) were affected by acute lymphoblastic leukemia (ALL), and 75 patients (92.5%) had non‐Hodgkin lymphoma (NHL). Of the NHL patients, 54 (73.0%) were categorized as DLBCL and 21 as non‐DLBCL. Treatment included Axicabtagene Ciloleucel for 39 patients (48%), Tisagenlecleucel for 25 patients (30%), and Brexucabtagene Autoleucel for 17 patients (22%). The comorbidities of the study population (Table 1) were categorized as follows: cardiovascular: 46/81 (56.8%); gastrointestinal: 23/81 (28.4%); smokers: 6/81 (7.4%); obesity: 5/81 (6.2%); endocrine: 13/81 (16.0%); infectious: 25/81 (30.9%); autoimmune: 11/81 (13.6%). Cardiovascular comorbidities mainly included arterial hypertension, which was well‐controlled with treatment. Gastrointestinal comorbidities included antral gastropathy and inflammatory bowel disease. Hepatic conditions included steatosis, previous viral hepatitis under prophylaxis, and cholelithiasis. Autoimmune comorbidities were limited to single‐organ manifestations, not requiring continuous immunosuppression (primarily affecting the thyroid and skin). Endocrine comorbidities included thyroid function impairment managed with supplementation in 12 cases, while one patient had secondary hypoparathyroidism. Despite the prevalence of these comorbidities, all patients were deemed fit for chemotherapy by a multidisciplinary team. The cohort had received multiple prior treatments, ranging from 2 to 9 lines (median: 3.4). Rituximab was included in the treatment plan for 51 out of 81 patients (63.0%). Patients were stratified by exposure to infections based on IgG‐positive serologies recorded during hospitalization before CAR‐T cell infusion. The results were SARS‐CoV‐2: 15/81 (18.5%); Hepatitis A virus: 11/81 (13.6%); Hepatitis B virus: 9/81 (11.1%); Herpes simplex virus: 12/81 (14.8%); Cytomegalovirus: 11/81 (13.6%); Epstein–Barr virus: 13/81 (16.0%). Low prevalence of positive serologies may be attributed to prior anti‐B lymphocyte treatments. Additionally, 23 out of 81 patients (28.4%) had previously received intrathecal chemotherapy, with an average of 6.4 punctures per patient.

**TABLE 1:** Patients’ demographic and clinical characteristics.

Demographics and clinical characteristics baseline comparison	Total Cohort (*n* = 81)	ICANS + (*n* = 26)	ICANS − (*n* = 55)
Age (years), mean ± SD	56.20 ± 13.03	61.00 ± 10.71	59.93 ± 13.49
Sex			
Females, *n*(%)	35 (43.2%)	8 (9.9%)	27 (33.3%)
Males, *n*(%)	46 (56.8%)	18 (22.2%)	28 (34.6%)
Comorbidities			
Smokers, *n*(%)	6 (7.4%)	1 (3.8%)	5 (9.0%)
Cardiovascular, *n*(%)	46 (56.8%)	16 (61.5%)	30 (54.5%)
Gastrointestinal, *n*(%)	23 (28.4%)	5 (19.2%)	18 (32.7%)
Obesity, *n*(%)	5 (6.2%)	1 (3.8%)	4 /7.2%)
Endocrine, *n*(%)	13 (16.0%)	3 (11.5%)	10 (18.1%)
Infectious, *n*(%)	25 (30.9%)	9 (34.6%)	16 (29.0%)
Autoimmune, *n*(%)	11 (13.6%)	4 (15.3%)	7 (12.7%)
Epilepsy history, *n*(%)	1 (1.2%)	1 (3.8%)	0 (0%)
Histopathology			
DLBC, *n*(%)	54 (66.7%)	17 (65.0%)	37 (67.0%)
Non‐DLBC, *n*(%)	27 (33.3%)	9 (34.6%)	18 (32.7%)

Abbreviations: DLBC (Diffuse Large B‐cell Lymphoma); ICANS (Immune Effector Cell‐associated Neurotoxicity Syndrome).

### 3.2 CRS and ICANS Characteristics

After CAR‐T infusion, we observe hematological alterations and biochemical abnormalities (see Table S1). In detail, all patients had anemia (< 13 g/dL); thrombocytopenia (< 150 × 10⁹/L) was observed in 64/81 patients, leukopenia (< 4 × 10⁹/L) in 71/81 (87.7%), neutropenia (< 1.8 × 10⁹/L) in 57/81 (70.4%), and lymphopenia (< 1 × 10⁹/L) in 58/81 (71.6%). Elevated gamma‐GT (> 70 U/L) was noted in 22/81 (27.2%), LDH (> 250 U/L) in 20/81 (24.7%), and GPT in 5/81 (6.2%). CRS developed in 76 out of 81 patients (93.8%), with an average onset of 2.4 days posttreatment. CRS severity was categorized as Grade 1: 28 patients (34.6%); Grade 2: 35 patients (43.2%), Grade 3–4: 13 patients (16.0%). Key symptoms included hyperpyrexia (91.4%), hypoxia (39.5%), and hypotension (37.0%). ICANS was observed in 26 patients (32.1%). The average onset was 5.5 days posttreatment, with a mean ICANS grade of 2.0. All ICANS+ patients had prior CRS (median grade: 2). Among ICANS+ patients, major neurological symptoms included dysgraphia (42.3%), tremor (23.1%), aphasia (15.4%), and seizures (15.4%). As expected, ICANS occurred less frequently in patients treated with 4‐1BB co‐stimulated CAR‐T cells compared to CD28 co‐stimulated CAR‐T cells (39% vs. 19%, *p* = 0.107).

### 3.3 Electrophysiological Findings

Before CAR‐T infusion, a baseline standard EEG was performed in 68 patients. Interestingly, we found a significantly higher prevalence of focal‐to‐bilateral slowing activity and focal slowing activity at baseline in patients who developed ICANS compared to those who did not (Table 2).

**TABLE 2:** Baseline and acute phase EEG findings: statistical comparison between ICAN positive (ICAN+) and ICAN negative (ICAN−) groups of patients.

Baseline EEG findings comparison	Total cohort (*n *= 68)	ICANS + (*n *= 23)	ICANS − (*n *= 45)	*p*	Baseline EEG findings comparison	Total cohort (*n *= 36)	ICANS + (*n *= 24)	ICANS − (*n *= 12)	*p*
EEG normal/not normal					EEG normal/not normal				
Normal, *n*(%)	45 (66.2%)	8(11.8%)	37 (54.4%)	**< 0.001**	Normal, *n*(%)	3 (8.3%)	0(0%)	3 (25%)	**< 0.031**
Not normal, *n*(%)	23 (33.8%)	15 (22.1%)	8 (17.8%)		Not normal, *n*(%)	33 (91.7%)	24 (66.7%)	9(25%)	
Diffuse slow (Theta and Delta) activity, *n*(%)	11 (47.8%)	6 (8.8%)	5 (7.4%)	0.164	Diffuse slow (Theta and Delta) activity, *n*(%)	25(69.4%)	19(52.8%)	6 (16.7%)	0.124
Focal slow activity, *n*(%)	7 (10.3%)	6 (8.8%)	1 (1.5%)	**0.005**	Focal slow activity, *n*(%)	7 (19.4%)	7 (19.4%)	0 (0%)	0.07
Focal synchronous bilateral slow activity, *n*(%)	14 (20.8%)	11 (16.2%)	3 (4.4%)	**< 0.001**	Focal synchronous bilateral slow activity, *n*(%)	17 (47.2%)	12(33.3%)	5 (13.9%)	0.732
Generalized (synchronous) epileptiform discharges, *n*(%)	0 (0%)	0 (0%)	0 (0%)	NA	Generalized (synchronous) epileptiform discharges, *n*(%)	1 (2.8%)	1 (2.8%)	0 (0%)	0.473
Focal epileptiform discharges, *n*(%)	0 (0%)	0 (0%)	0 (0%)	NA	Focal epileptiform discharges, *n*(%)	6 (16.7%)	6 (16.7%)	0 (0%)	0.079
PDR pre, mean ± SD	9.37 ± 1.04	9.13 ± 1.20	9.49 ± 0.94	0.143	PDR pre, mean ± SD	7.33 ± 1.96	7.15 ± 1.86	7.67 ± 2.19	0.344

Abbreviations: EEG, electroencephalogram; PDR, posterior dominant rhythm.

**FIGURE 1:**
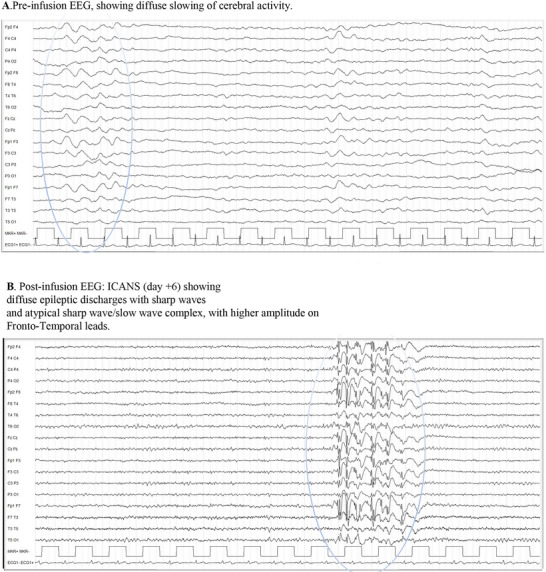
(A) Pre‐infusion EEG showing diffuse slowing of cerebral activity (B) Post‐infusion EEG: ICANS (Day +6) showing diffuse epileptic discharges with sharp waves and atypical sharp wave/slow wave complex with higher amplitude in fronto‐temporal leads.

EEG abnormalities were noted in all patients developing ICANS, primarily consisting of diffuse slow‐wave activity (theta and delta waves) and/or focal synchronous bilateral slow activity (Table 2). Intriguingly, EEG has proven to be highly sensitive in the early detection of neurological toxicity: even in patients presenting only mild dysgraphia (ICE score 9/10), the appearance of slow‐wave activity on EEG was a strong predictor of ICANS, guiding also therapeutic decisions (Figure 1).

Moreover, two patients with ICANS developed nonconvulsive status epilepticus (NCSE), treated with levetiracetam (3 or 2 g/day IV). One patient experienced focal to bilateral tonic–clonic seizures (FBTCS), managed with diazepam and valproic acid (1600 mg IV infusion). One patient had refractory status epilepticus (RSE), treated with diazepam and valproic acid.

### 3.4 Predictive Biomarkers

Baseline hematological parameters and patients’ characteristics showed significant differences between ICANS+ and ICANS− patients. Baseline higher age (*p* = 0.025), higher gammaGT (*p* = 0.003), higher CRP (*p* = 0.036), higher alkaline phosphatase (*p* = 0.037), lower PLT count (*p* = 0.016), and higher mEASIX (*p* = 0.035) were found in ICANS+ patients (see Table S2). Patients developing ICANS were found to experience deeper anemia (*p* = 0.027), leukopenia (*p* = 0.011), and maintain higher gamma‐GT (*p* = 0.003), compared to ICANS− patients (see Table S1).

**FIGURE 2:**
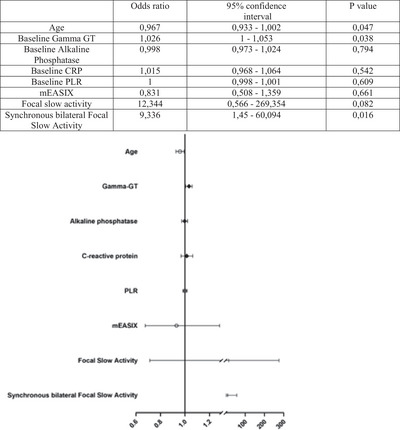
Multivariate statistical analysis. The table shows odds ratios, confidence intervals and *p* values for each variable highlighted in the forest plot.

A multivariate analysis identified younger age as protective against ICANS (OR = 0.964, *p* = 0.047), while elevated GammaGT (cut‐off 45) (OR = 1.026, *p* = 0.038) and focal synchronous bilateral slow EEG activity (OR = 9.336, *p* = 0.016) were significant predictors of ICANS (Figure 2). Among the 10 patients with both baseline diffuse cerebral slowing and elevated GammaGT (> 45), 9 developed ICANS (OR = 9, 95% CI 1.14–71.03).

**FIGURE 3:**
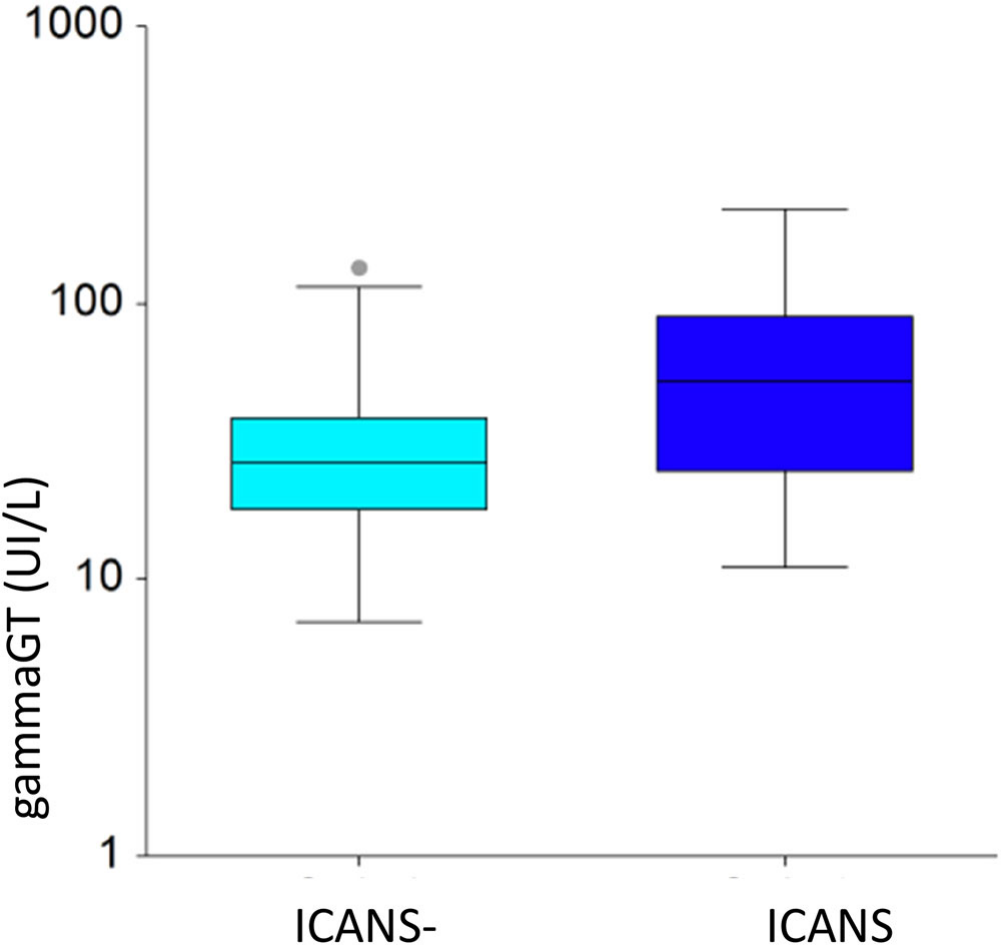
Box‐and‐whisker plot to show the statistically significant correlation between GammaGT values and the development of ICANS (*p *= 0.00048).

There is a significant overlap in GammaGT values between individuals who develop ICANS and those who do not. We then performed an ROC analysis to determine a cut‐off value for GammaGT to better stratify the ratio (Figure S1). We found that a cut‐off of 45 UI/L predicted ICANS with a sensitivity of 58% and a specificity of 83%. (Figure 3)

## 4 Discussion

The prevalence of ICANS in our sample was 32%, consistent with data reported in the literature (Grant et al. 2022; Siegler and Kenderian 2020). Moreover, the study on acute‐phase markers in ICANS+ patients reveals robust findings that align with established hypotheses and current literature (Galli et al. 2023). Regarding demographic findings, we observed that patients developing ICANS after treatment were significantly older compared to those without neurotoxicity. Previous literature indicated that younger age is associated with a higher risk of neurotoxicity, as ICANS incidences appear to be higher in pediatric and young adult populations compared to older adults (Hunter and Jacobson 2019). This difference can be attributed to the gradual maturation of astrocytes after birth, making them more susceptible to injury and compromise of the neurovascular unit (NVU). Consequently, the blood–brain barrier is more vulnerable in younger populations. (Molofsky and Deneen 2015). However, this trend seems to be reversed in adult samples, as extensively described in current literature (Grant et al. 2022). This is most likely due to a multifaceted process called “inflammaging,” which refers to the cumulative outcome of chronic physiological stimulation of the innate immune system caused by the accumulation of cellular damage, chronic conditions, reduced anti‐inflammatory response, and lifestyle changes. This scenario can become detrimental with age, creating an ideal environment for the onset of several conditions rooted in inflammatory mechanisms, potentially including CAR‐T‐related neurotoxicity (Franceschi et al. 2018). In fact, our results confirm that age is positively correlated with ICANS development in the adult population. The mechanism underlying ICANS development has been widely debated in the scientific community. However, there is broad consensus, though lacking definitive evidence, that neurotoxicity following CAR‐T therapy is caused by an inflammatory cascade driven by myeloid cell‐derived cytokines, most notably IL‐1 and IL‐6. This could lead to increased endothelial activation, systemic capillary leakage, and subsequent permeability of the blood–brain barrier (Wang et al. 2025; Siegler and Kenderian 2020). In our population, and consistent with the hypothesized inflammatory pathophysiology, CRP, basophils, and the mEASIX score ([LDH (U/L) × CRP (mg/dL)]/PLTs (10⁹ cells/L)) are positively correlated with ICANS development. Interestingly, in our sample, patients who developed ICANS had higher baseline levels of gamma‐GT compared to those who did not. This is the first report of such findings, both before and after the development of neurotoxicity. In other words, it appears that altered hepatic function might be a predisposing factor for ICANS, in addition to characterizing the acute phase of the condition. A possible explanation for this difference is that patients who develop ICANS might have been treated for malignancy with more hepatotoxic drugs. However, we did not find any differences between the two groups in terms of the number or type of previous treatments.

Thus, we can hypothesize that an altered metabolic profile might represent another predisposing factor for the development of neurotoxicity, in addition to being a clinical expression of the condition. Recent studies have demonstrated that gamma‐GT also plays an important role in neuroinflammation: gamma‐GT‐mediated cleavage of glutathione promotes iron redox cycling, stimulating the release of hydroxyl radicals by microglia and infiltrating macrophages in a mouse model of CNS inflammation as well as in human multiple sclerosis lesions (Mendiola et al. 2020). Therefore, gamma‐GT might be considered both an easily detectable predictor and an inexpensive marker of ICANS. Conversely, other important inflammatory parameters, such as fibrinogen and ferritin—widely reported as specific inflammatory markers for ICANS—do not correlate at baseline with neurotoxicity development in our sample. Additionally, the PLR was observed to be lower at baseline in patients who developed ICANS.

PLR is a novel systemic inflammatory marker that has been investigated as a potential reliable prognostic factor for patients with malignancy. In recent literature, PLR has been described as both a positive and a negative predictive marker for overall survival in cancer patients (Zheng et al. 2017). In our study, we recorded a negative association between PLR values and ICANS development. The presence and severity of CRS have been extensively associated with ICANS development in the literature (Grant et al. 2022). In our population, all 26 ICANS patients had developed CRS, but the percentage of patients with CRS was not significantly different in ICANS‐ patients. Therefore, CRS and ICANS seem to be two distinct and unrelated consequences of a common systemic pro‐inflammatory background. Regarding neurophysiological predictors, we found a significantly higher prevalence of focal‐to‐bilateral slowing activity and focal slowing activity at baseline in patients who developed ICANS compared to those who did not. This type of EEG abnormality is widely recognized as a typical expression of metabolic encephalopathy. (Motomura et al. 2012) Supporting this, Kim et al. (2021) demonstrated that the majority of such anomalies (specifically FIRDA) without structural brain lesions were attributable to a combination of metabolic encephalopathy and neurodegenerative disease. These findings align with the hypothesis of the role of metabolic abnormalities in the pathophysiology of neurotoxicity.

In conclusion, our study confirmed the critical role of baseline EEG abnormalities as predictors of CAR‐T neurotoxicity. Additionally, we identified gamma‐GT as a new predictive parameter in the pathophysiology of ICANS.

More specifically, we identified a small group of patients at very high risk, suitable for tailored clinical monitoring. Since antiepileptic prophylaxis is still under debate, we may speculate, for example, that this cohort of patients could benefit from antiepileptic prophylaxis.

### 4.1 Study Limitations

The retrospective design and single‐center setting of our study limit external validity. Clearly, this represents only an exploratory study, and our model would then need to be tested on new groups of patients. Further research is necessary to validate our findings so that they can eventually be integrated into clinical practice.

## Author Contributions

**Anna Modoni**: conceptualization, supervision, writing – original draft. **Catello Vollono**: data curation, software. **Eugenio Galli**: formal analysis, methodology. **Luca Capriati**: data curation. **Federica Sorà**: supervision. **Stefan Hohaus**: supervision. **Serenella Servidei**: validation. **Nicola Piccirillo**: investigation. **Paolo Calabresi**: validation. **Simona Sica**: conceptualization, supervision.

## Conflicts of Interest

The authors declare no conflicts of interest.

## Peer Review

The peer review history for this article is available at https://publons.com/publon/10.1002/brb3.70891.

## Supporting information




**Supplementary Figure**: brb370891‐sup‐0001‐FigureS1.docx

**Supplementary Table**: brb370891‐sup‐0002‐TableS1.docx

**Supplementary Table**: brb370891‐sup‐0003‐TableS2.docx

## Data Availability

The data that support the findings of this study are available on request from the corresponding author. The data are not publicly available due to privacy or ethical restrictions.
