# Supplementary figures and images for: Predictors of Neurotoxicity in a Large Cohort of Italian Patients Undergoing Anti‐CD19 Chimeric Antigen Receptor (CAR) T‐Cell Therapy
Source: Brain Behav. 2025 Sep 23;15(9):e70891. doi: 10.1002/brb3.70891 (PMC12455013; doi:10.1002/brb3.70891)

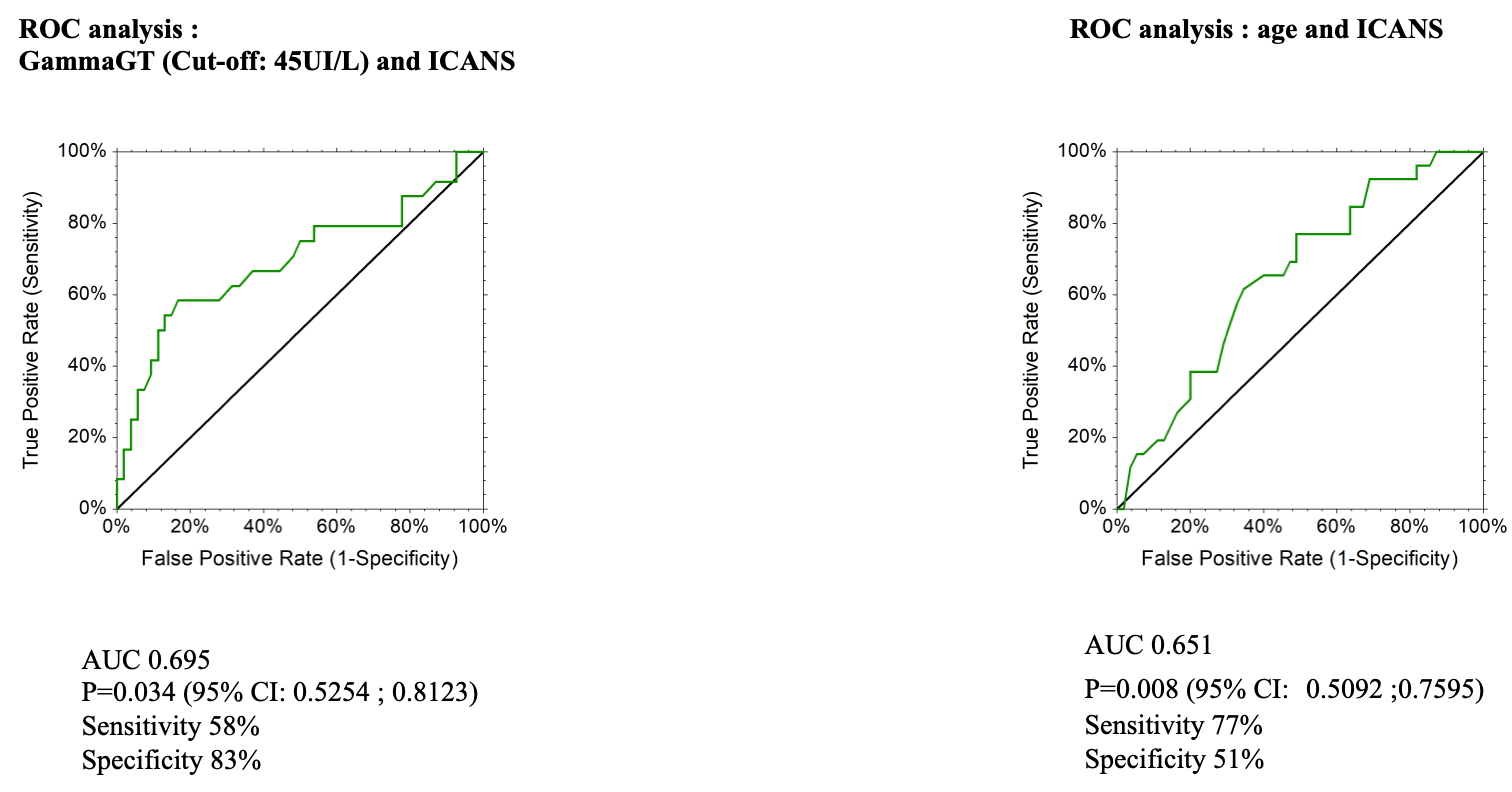


**Supplementary figure:** ROC analysis

Supplement: Supplementary file 1 — Supplementary Figure: brb370891‐sup‐0001‐FigureS1.docx [file BRB3-15-e70891-s001.docx]
