# Supplementary material for: Predictors of Neurotoxicity in a Large Cohort of Italian Patients Undergoing Anti‐CD19 Chimeric Antigen Receptor (CAR) T‐Cell Therapy
Source: Brain Behav. 2025 Sep 23;15(9):e70891. doi: 10.1002/brb3.70891 (PMC12455013; doi:10.1002/brb3.70891)
Supplement: Supplementary file 2 — Supplementary Table: brb370891‐sup‐0002‐TableS1.docx [file BRB3-15-e70891-s002.docx]

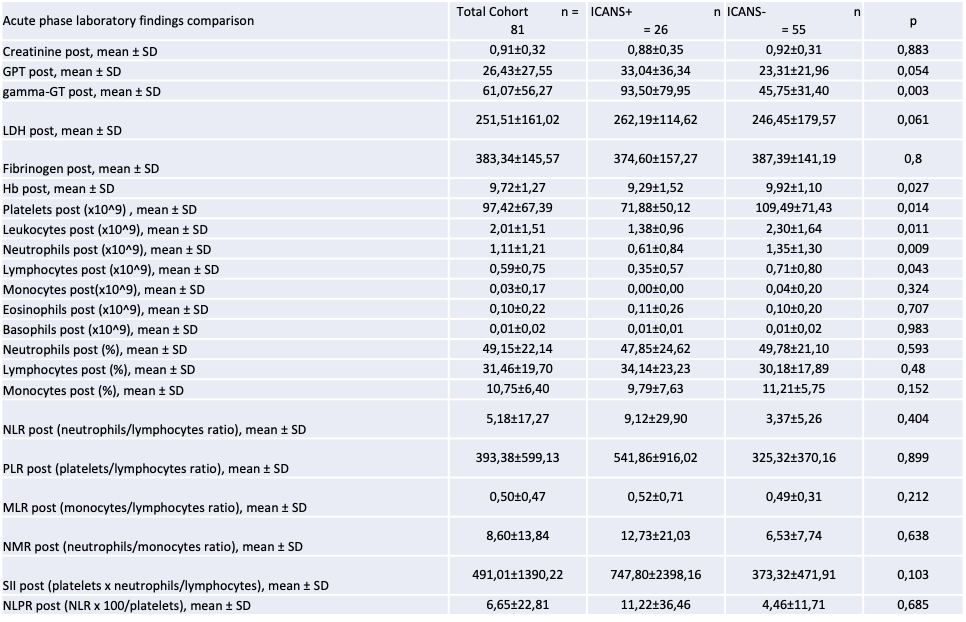


**SUPPLEMENTARY TABLE 1**: Statistical comparison of laboratory findings measured between day 0 and 14 (“acute phase”) between ICANS-positive (ICANS+) and ICANS-negative (ICANS−) patient groups. The acute phase refers to the numerical values at their maximal deviation from the normal range within the first month following therapy.

Abbreviations: GPT (Glutamic Pyruvic Transaminase); GammaGT (Gamma Glutamyl Transpeptidase); LDH (Lactic Dehydrogenase); HB (Hemoglobin)
