# Supplementary material for: Predictors of Neurotoxicity in a Large Cohort of Italian Patients Undergoing Anti‐CD19 Chimeric Antigen Receptor (CAR) T‐Cell Therapy
Source: Brain Behav. 2025 Sep 23;15(9):e70891. doi: 10.1002/brb3.70891 (PMC12455013; doi:10.1002/brb3.70891)
Supplement: Supplementary file 3 — Supplementary Table: brb370891‐sup‐0003‐TableS2.docx [file BRB3-15-e70891-s003.docx]

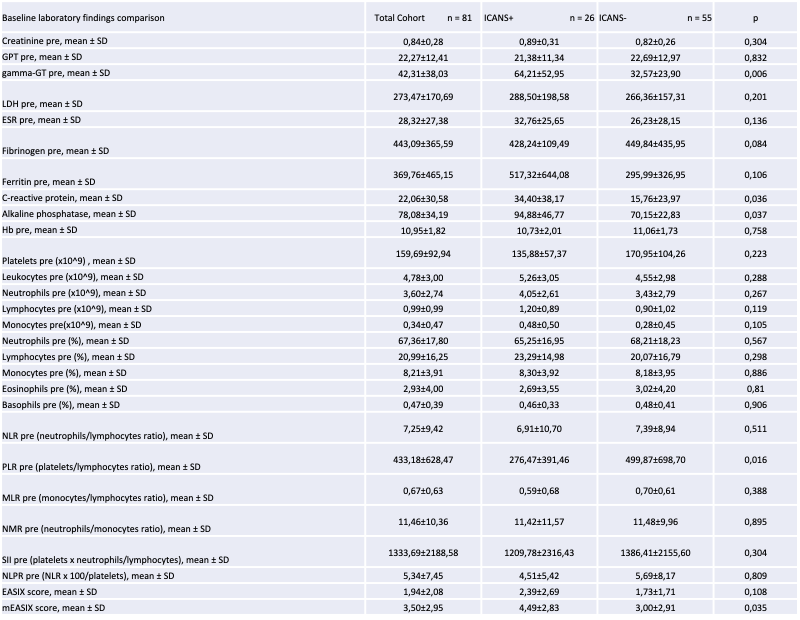


**Supplementary table 2**: Statistical comparison of baseline findings between ICANS-positive (ICANS+) and ICANS-negative (ICANS−) patient groups. Abbreviations: GPT (Glutamic Pyruvic Transaminase); GammaGT (Gamma Glutamyl Transpeptidase); LDH (Lactic Dehydrogenase); HB (Hemoglobin)
